# Implementation of Departmental Quality Strategies Is Positively Associated with Clinical Practice: Results of a Multicenter Study in 73 Hospitals in 7 European Countries

**DOI:** 10.1371/journal.pone.0141157

**Published:** 2015-11-20

**Authors:** Rosa Sunol, Cordula Wagner, Onyebuchi A. Arah, Solvejg Kristensen, Holger Pfaff, Niek Klazinga, Caroline A. Thompson, Aolin Wang, Maral DerSarkissian, Paul Bartels, Philippe Michel, Oliver Groene, DUQuE Project Consortium

**Affiliations:** 1 Avedis Donabedian Research Institute (FAD), Universitat Autonoma de Barcelona, Barcelona, Spain; 2 Red de investigación en servicios de salud en enfermedades crónicas REDISSEC, Barcelona, Spain; 3 NIVEL, Netherlands Institute for Health Services Research, Utrecht, The Netherlands; 4 Department of Public and Occupational Health,EMGO Institute for Health and Care Research, VU University Medical Center, Amsterdam, The Netherlands; 5 Department of Epidemiology, Fielding School of Public Health, University of California Los Angeles (UCLA), Los Angeles, California, United States of America; 6 Danish Clinical Registries, Aarhus, Denmark; 7 Department of Health Science and Technology, Aalborg University, Aalborg, Denmark; 8 Institute for Medical Sociology, Health Services Research and Rehabilitation Science, University of Cologne, Cologne, Germany; 9 Center for Health Services Research Cologne, University of Cologne, Cologne, Germany; 10 Department of Public Health, Academic Medical Center, University of Amsterdam, Amsterdam, The Netherlands; 11 Palo Alto Medical Foundation Research Institute (PAMFRI), Palo Alto, California, United States of America; 12 Hospices Civils de Lyon, Université Claude Bernard Lyon 1, Lyon, France; 13 Department of Health Services Research and Policy, London School of Hygiene and Tropical Medicine, London, United Kingdom; University of Florence, ITALY

## Abstract

**Background:**

Given the amount of time and resources invested in implementing quality programs in hospitals, few studies have investigated their clinical impact and what strategies could be recommended to enhance its effectiveness.

**Objective:**

To assess variations in clinical practice and explore associations with hospital- and department-level quality management systems.

**Design:**

Multicenter, multilevel cross-sectional study.

**Setting and Participants:**

Seventy-three acute care hospitals with 276 departments managing acute myocardial infarction, deliveries, hip fracture, and stroke in seven countries.

**Intervention:**

None.

**Measures:**

Predictor variables included 3 hospital- and 4 department-level quality measures. Six measures were collected through direct observation by an external surveyor and one was assessed through a questionnaire completed by hospital quality managers. Dependent variables included 24 clinical practice indicators based on case note reviews covering the 4 conditions (acute myocardial infarction, deliveries, hip fracture and stroke). A directed acyclic graph was used to encode relationships between predictors, outcomes, and covariates and to guide the choice of covariates to control for confounding.

**Results and Limitations:**

Data were provided on 9021 clinical records by 276 departments in 73 hospitals. There were substantial variations in compliance with the 24 clinical practice indicators. Weak associations were observed between hospital quality systems and 4 of the 24 indicators, but on analyzing department-level quality systems, strong associations were observed for 8 of the 11 indicators for acute myocardial infarction and stroke. Clinical indicators supported by higher levels of evidence were more frequently associated with quality systems and activities.

**Conclusions:**

There are significant gaps between recommended standards of care and clinical practice in a large sample of hospitals. Implementation of department-level quality strategies was significantly associated with good clinical practice. Further research should aim to develop clinically relevant quality standards for hospital departments, which appear to be more effective than generic hospital-wide quality systems.

## Introduction

Substantial research has been undertaken on the assessment and improvement of the quality of health care delivery in the past 30 years. While considerable progress has been made, quality and safety problems persist and the debate on how to accelerate and sustain quality management is more relevant than ever [1–4]. In response to this debate, a new research line related to the effectiveness of quality management has emerged in the last 10 to 15 years, with a focus on questions such as "Does quality management lead to better quality of care?", "Which quality tools are most effective?", and "What factors are associated with the effective implementation of quality management systems?” [5–11].These questions are highly relevant for hospital managers and clinicians aiming to implement quality management systems targeting organization-specific quality and safety issues.

Quality management systems can be understood as sets of interacting activities, methods, and procedures used to direct, monitor, control, and improve quality of care [12]. They usually exist at the highest levels of the organization and are operationalized into specific quality improvement activities within smaller organizational units. It is assumed that quality management systems are a prerequisite for the systematic application and sustainability of quality improvement activities through smaller units, which are needed to reduce undesired variations in clinical practice and to improve the effectiveness, safety, and patient-centeredness of care [13].

While variations in clinical practice are well documented, there is little evidence on factors associated with the uptake of quality management activities by hospitals or on their impact on clinical practice. A recent systematic review identified a number of studies exploring factors positively associated with the implementation of quality management systems [14]. Previous reports have identified positive associations between engagement in quality improvement activities and a range of perceived outcomes (e.g., patient satisfaction, productivity, and physician-nurse relations) and quality improvement outputs (e.g., use of ID bracelets) [6, 13, 15]. However, none of these studies have investigated the impact of quality management systems on clinical practice. Our study seeks to address this gap.

The study was conducted as part of the “Deepening our understanding of quality improvement in Europe (DUQuE)” project, which was funded by the European Union’s Seventh Research Framework Program. The overall aim of the project was to study relationships between organizational quality improvement systems and organizational culture, professional involvement, and patient empowerment at hospital and department levels, and to analyze the quality of health care delivery in terms of clinical effectiveness, patient safety, and patient experiences. In previous work, we explored the effects of hospital-level quality management systems on quality activities at the department level, and found mixed results, with positive but weak associations [16]. Our specific objectives in the present study were to assess performance based on clinical practice indicators and to explore their association with the implementation of hospital and departmental quality management systems in 4 clinical settings in a large sample of European hospitals.

## Methods

### Study Design, Setting and Population

In this multicenter cross-sectional study, data were collected at the hospital-, department-, and patient-level through questionnaires administered to managers and health professionals, retrospective case note reviews, and direct observation. Hospitals with more than 130 beds were randomly selected in each the participating countries: Czech Republic, France, Germany, Poland, Portugal, Spain, and Turkey. Including criteria was to reach general acute care hospitals, with a minimum hospital size of 130 beds that had a sufficient volume of care to ensure recruitment of 30 patients per condition over a 4-month period (a sample frame of a minimum of 90 patients). Specialty hospitals, hospital units within larger hospitals, and hospitals not providing care for the four clinical conditions of study were excludedn each hospital, detailed information was collected on 4 conditions: acute myocardial infarction, vaginal deliveries, hip fractures, and stroke. For each condition, we reviewed 35 consecutive cases that fulfilled the study’s inclusion and exclusion criteria study. Data for the clinical practice indicators were retrieved through retrospective case note review using a standardized data collection sheet and a manual translated into the local language. The review of case notes was organized locally according to protocol and performed by trained hospital staff with clinical background knowledge and experience with local clinical and documentation practice and not being involved in the practice being assessed. Other department’s data was retrieved from the department seeing most patients from the studied condition and emergency area (if applicable). All data were collected between May 2011 and February 2012. The data collection strategy was informed by a sample size calculation that took into account the multilevel structure of the study [17]. The broader theoretical conceptual framework, detailed objectives and methods, and different constructs assessed within the DUQuE project are described in detail elsewhere [18].

### Outcomes, Predictors and Covariates

The outcome variables consisted of a set of 19 single clinical practice indicators selected based on a review of the literature and a systematic rating procedure by individual experts and relevant European scientific societies to assess their relevance, applicability, and feasibility. In addition, 5 clinical practice summary measures were constructed (see Textbox 1). Each clinical indicator was assigned a level of evidence rating (A, B, C, etc.) based on the scientific evidence quoted in the pertinent clinical practice guidelines [18].

**Textbox 1. Clinical practice summary indicators and predictor variables (measuring the implementation of quality management systems at the hospital and department levels)**

Clinical practice summary indicators*

- *Acute AMI*: a) Was **reperfusion therapy given on time** (fibrinolytic agent administered within 75 minutes of hospital arrival or primary percutaneous coronary intervention within 90 minutes)?; b) Were **all appropriate medications** (including anti-platelet drugs, beta-blockers, statin and ACE inhibitors) prescribed (or contraindicated) at discharge?
- *Routine vaginal deliveries*: Were there any **birth-related complications** (blood transfusion, acute C-section (planned vaginal delivery that finished in a C-section), instrumentation needed during vaginal delivery, third- or fourth-degree laceration, or newborn Apgar score <7 at 5 minutes)?
- *Hip fractures*: What **percentage of recommended care was delivered?** (This was calculated from the following measures: prophylactic antibiotic within 60 minutes prior to surgical incision, prophylactic thrombolytic treatment received on the same day of admission, patient mobilized within 24 hours after surgery and patients with in-hospital surgical waiting time <48 hours.)
- *Stroke*: **Did stroke patients receive appropriate care?**: (Appropriate care included treatment with an antiplatelet inhibitor within 48 hours, performance of a CT or MRI within 24 hours, and mobilization of the patient within 48 hours.)

*Clinical practice summary indicators had 3 possible values: “Yes”, “No”, or “Not applicable”.

Predictors at the hospital level**

• ***Quality Management Systems Index* (QMSI).** The QMSI is an overall measure of the extent of implementation of quality management systems and has 9 dimensions: 1) quality policy documents, 2) quality monitoring by hospital board, 3) training of professionals, 4) formal protocols for infection control, 5) formal protocols for medication and patient handling, 6) analysis of performance of care processes, 7) analysis of performance of professionals, 8) analysis of patient feedback, and 9) evaluation of results.
- ***Quality Management Compliance Index* (QMCI)**. The QMCI is a measure of how the hospital management oversees hospital quality program initiatives and has 4 dimensions: 1) quality planning, 2) monitoring of patient/professional opinions, 3) monitoring of quality systems, and 4) improvement of quality through staff development activities.
- ***Clinical Quality Implementation Index* (CQII).** The CQII measures the implementation of quality efforts throughout the hospital and analyzes continuous improvement in clinical areas with 7 dimensions: 1) prevention of hospital infections, 2) medication management, 3) prevention of patient falls, 4) prevention of patient ulcers, 5) routine testing of elective surgery patients, 6) safe surgical practices, and 7) prevention of patient deterioration.

**All quality management measures are rated from 0 (lowest possible score) to 10 (highest possible score). Methods for the construction of all measures, including results from the factor analysis and rationale for the dimensions selected are provided in detail elsewhere [19–21].

Predictors at the department level***

- **Specialized expertise and responsibility (SER**). This assesses professional expertise and the allocation of clinical responsibilities. [21]
- **Evidence-based organization of pathways (EBOP)**. This assesses the extent to which departmental organizational processes incorporate evidence-based care recommendations (as expressed in NICE quality standards and SIGN audit tools) [22–26].
- **Patient Safety Strategies (PSS).** These identify the application of strategies for ensuring patient safety recommended by international agencies including patient identification, hand hygiene, medication management, resuscitation processes and adverse event declaration [21, 26]
- **Clinical Review (CR).** This assesses the use of clinical audit and systematic monitoring as part of department-level quality management activities. [21]

***All department quality measures were rated on a 5-point Likert scale and were common to all conditions, except EBOP, which was based on specific evidence findings for each condition.

Predictor variables measuring characteristics of quality management system implementation included 3 quality index scores at the hospital level and 4 quality measures at the department level that cover complementary aspects of quality management systems. These measures were build on previously validated tools, and their detailed (psychometric) properties are described elsewhere [19–21] (see Textbox 1). One of the measures (the Quality Management Systems Index) was derived from a questionnaire administered to hospital quality managers. The other 6 measures were collected through direct observation by an external trained surveyor.

Additional variables collected and included in multivariate analyses included the country in which the hospital was based, hospital teaching status (teaching versus non-teaching), hospital size (<200, 200–500, 501–1000, or >1000 beds), hospital ownership (public versus private), and the gender and age of each patient included in the chart review population.

### Hypothesis and analytical strategy

We hypothesized that the implementation of hospital-level quality management systems and department quality activities (positively) predicted on clinical practice for all 4 conditions analyzed (acute AMI, vaginal deliveries, hip fractures, and stroke). We first described the hospitals in the sample, the mean scores for hospital- and department-level quality measures, the characteristics of the patients in the chart review population, and compliance with clinical indicators for each pathway analyzed. For categorical variables we calculated frequencies and percentages. For continuous variables, we calculated the mean and standard deviation. We provide details on missing data in the descriptive tables, but multivariable analyses excluded patients with incomplete records for any of the variables (exposure, confounders, and clinical practice indicators).

**Fig 1:**
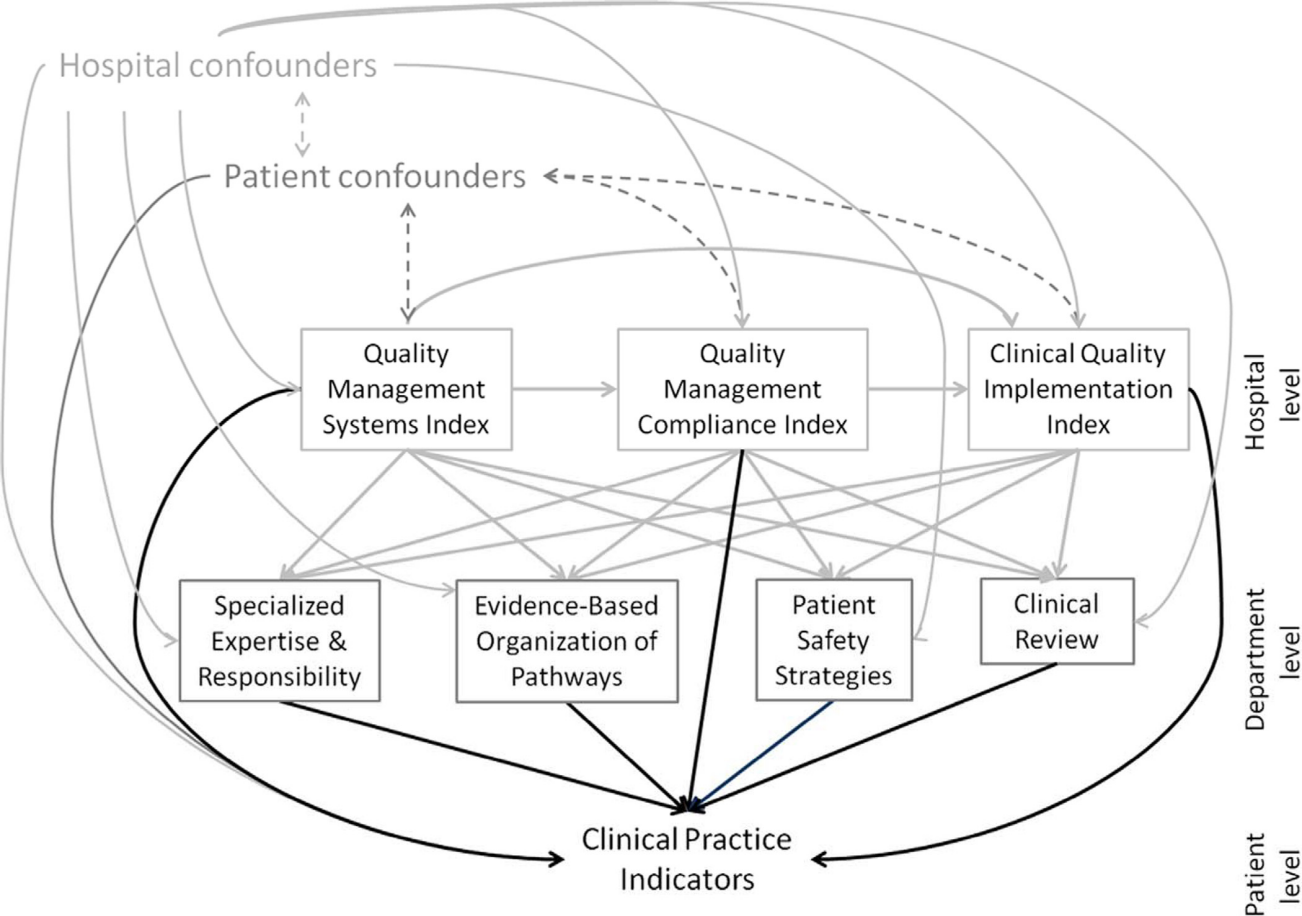
Directed acyclic graph (DAG) guiding the analysis and showing hypothesized relationships between predictors, outcome, and covariates in this study. Directed acyclic graph (DAG) used to guide the analysis and showing hypothesized relationships between predictors, outcome, and covariates in this study. Unidirectional arrows show an effect and bidirectional dashed arrows show a correlation. Black unidirectional arrows show the relationships tested and quantified in this article, whereas the gray arrows show relationships between other variables that guided the choice of confounding variables to control for in the analyses.

A directed acyclic graph (DAG) was used to depict our knowledge and assumptions about the (plausible) relations between predictors: hospital quality management measures, quality activities at the department level, and clinical practice indicators. Variable selection for the statistical models in this paper was guided by the DAG shown in Fig 1.

The edges in this graph encode relationships between predictors, outcomes, and covariates, and are governed by rules that affect choice of covariates to control for confounding [27–29]. For example, because we assumed that QMSI predicted QMSCI, when estimating the relationship between QMSCI and a patient-level measure, we additionally controlled for QMSI, as it would be a confounding variable for the relation of interest.

We estimated multivariate binomial logistic models with random intercept by hospital to account for clustering of patients within hospitals for all clinical indicators except percentage of recommended care delivered in the hip fracture pathway, for which we used multivariate ordinal logistic mixed model (also with random intercept by hospital). Our models were adjusted for confounding fixed effects at the country level (country), hospital level (number of beds, teaching status, ownership), and patient level (age, gender, education level), in addition to quality measures as dictated by the DAG in Fig 1. Results of associations are presented in clustered forest plots for each type of hospital- or department-level quality management measure (exposure variables). All statistical analyses were carried out in SAS (version 9.3, SAS Institute Inc., Cary, NC, 2012) and the forest plots were created using R [30]

DUQuE fulfilled the ethic requirements for research projects as described in the 7^th^ framework of EU DG Research. Ethics approval was granted through the Bioethical Committee of the Department of Health of the Government of Catalonia, Spain on 12th February 2010, in writing by Dr Josep M Busquets, responsible for bioethics at the Department of Health. Data collection in each country complied with confidentiality requirements according to national legislation or standards of practice of that country. Patient records and other information was anonymized and de-identified prior to analysis.

## Results

Overall, 276 departments from 73 hospitals in 7 countries (87% of expected) provided valid data. The hospital and department characteristics are shown in Table 1. Most hospitals were public (*n* = 58, 79.4%) and almost half had a teaching function (*n* = 33, 45.2%)

**Table 1:** Characteristics of Hospitals That Participated in the Study.

Characteristic	*n*	(%)
All Hospitals	73	(100)
Czech Republic	12	(16.4)
France	11	(15.0)
Germany	4	(5.4)
Poland	12	(16.4)
Portugal	10	(13.6)
Spain	12	(16.4)
Turkey	12	(16.4)
Departments	276	(100)
Acute myocardial infarction	63	(22.8)
Stroke	69	(25.0)
Hip fracture	72	(26.0)
Deliveries	72	(26.0)
Teaching hospitals	33	(45.2)
Public hospitals	58	(79.4)
Approximate number of beds in hospital		
<200	7	(9.5)
200–500	22	(30.1)
501–1000	31	(42.4)
>1000	13	(17.8)

A total of 9021 clinical records (77% of expected) were analyzed. Their characteristics are shown in Table 2.

**Table 2:** Characteristics of Patients in the Chart Review.

Characteristics	All Departments		Acute Myocardial Infarction		Deliveries		Hip Fracture		Stroke	
Total number of cases (% of total)	9021	(100)	2019	(22.3)	2337	(25.9)	2288	(25.3)	2377	(26.3)
Sex *n* (%)										
Female	5754	(63.7)	589	(29.1)	2337	(100)	1701	(74.3)	1127	(47.4)
Age (years), Mean (SD)	62.4	(23.0)	64.9	(13.8)	26.7	(5.0)	81.3	(7.8)	70.8	(15.4)
Age not reported, *n* (%)	538	(6.0)	15	(0.7)	461	(19.7)	49	(2.1)	13	(0.5)

Table 3 shows descriptive statistics for the predictor variables, which reveal that departments dealing with hip fractures have the lowest implementation scores for quality management activities, with the exception of patient safety strategies. The highest variation in quality between departments was observed for evidence-based organization of pathways and clinical review.

**Table 3:** Descriptive Statistics for Hospital- and Department-Level Quality Management Measures.

	All Departments		Acute Myocardial Infarction		Deliveries		Hip fracture		Stroke	
	Mean (0–10)	(SD)	Mean (0–10)	(SD)	Mean (0–10)	(SD)	Mean (0–10)	(SD)	Mean (0–10)	(SD)
Predictors (level) (scale range = 0–10)										
Quality Management Systems Index (hospital level)	7.2	(1.5)								
Quality Management Compliance Index (hospital level)	6.4	(1.9)								
Clinical Quality Implementation Index (hospital level)	5.9	(2.1)								
Specialized Expertise and Responsibility (department level)			6.5	(3.0)	6.8	(2.7)	5.5	(2.2)	6.8	(3.0)
Evidence-Based Organization of Departments (department level)			8.0	(2.2)	9.3	(0.7)	5.8	(2.7)	7.5	(2.2)
Patient Safety Strategies (department level)			6.5	(1.2)	6.8	(1.5)	6.3	(1.2)	6.3	(1.5)
Clinical Review (department level)			5.5	(3.5)	6.0	(3.5)	3.8	(3.2)	4.8	(3.5)

Descriptive results for the clinical practice indicators are shown in Table 4. Mean compliance for single indicators was 79.8%, but rates varied considerably, for example from 42.7% for mobilization of hip fracture patients within 24 hours of surgery to 97.7% for prescription of anti-platelets at discharge in AMI. Although some level A evidence-based practice indicators seem to be fully implemented (e.g., anti-platelet prescription at discharge for AMI), performance was generally low in other areas, such as admission to a specialized stroke unit (or specific area) within 24 hours of arrival for stroke patients (compliance of 50.5%) or appropriate use of antibiotic or thrombotic prophylaxis in hip fracture patients (70.3% and 69.8% respectively). Analysis of compliance with the 5 clinical indicator summary measures showed compliance with two measures in approximately two-thirds of patients: appropriate prescription of all medication at discharge for AMI and complication-free births. Compliance was over 50% for the appropriate management of stroke and under 50% for 2 measures: administration of appropriate, timely reperfusion therapy for AMI patients (40%) and delivery of 75% of recommended care for hip fracture patients (30.7%). We also report in this table the range of country specific values for clinical indicators regarding their percentage of positive achievement

**Table 4:** Descriptive Statistics for Clinical practice Indicators (^*^).

CLINICAL practice indicators	Total Applicable Cases	Yes (%)		95% CI	Country Range^1^	Level of evidence	Source
**AMI (N = 2019)**							
Reperfusion therapy given	1998	1588	(79.5)	77.7–81.3	67.0–93.6	—	
**Reperfusion therapy given on time** (fibrinolytic agent administered within 75 min of hospital arrival or Primary percutaneous coronary intervention within 90 min)	1416	566	(40.0)	37.4–42.5	26.6–53.9	A and A/B	AHRQ
Anti-platelet prescribed (or contraindicated) at discharge	1809	1767	(97.7)	97.0–98.4	91.1–100	A	AHRQ
Beta blocker prescribed (or contraindicated) at discharge	1796	1605	(89.4)	87.9–90.8	83.1–95.0	A	AHRQ
Statin prescribed (or contraindicated)at discharge	1798	1632	(90.8)	89.4–92.1	80.5–96.1	A	AHRQ
ACE inhibitor prescribed (or contraindicated)at discharge	1765	1502	(85.1)	83.4–86.8	78.4–92.8	A	AHRQ
**Appropriate medications (anti-platelet, beta blocker, statin** *and* **ACE inhibitor) prescribed (or contraindicated) at discharge**	1778	1301	(73.2)	71.1–75.2	61.5–86.8		
**Deliveries (N = 2337)**							
Blood transfusion related to vaginal birth	1735	23	(1.3)	0.8–1.9	0.6–1.8	B	The Danish Clinical Registries
Acute C-section	2018	132	(6.5)	5.5–7.6	2.3–14.3	—	
Vaginal delivery with instrumentation	1893	244	(12.9)	11.4–14.4	0.6–24.7	B	OECD
Cases with 3rd or 4th degree laceration	2196	65	(3.0)	2.3–3.7	0.2–6.3	B	OECD
Adverse birth outcome (child): Apgar score below 7 at 5 minutes	2118	24	(1.1)	0.7–1.6	0.3–2.6	B	The Danish Clinical Registries
**Birth with complications (at least one of above indicators positive)**	1706	431	(25.3)	23.2–27.3	7.3–42.8		
**Hip fracture (N = 2288)**							
Prophylactic antibiotic within 60 minutes prior to surgical incision	1400	984	(70.3)	67.9–72.7	48.5–90.2	A	RAND
Prophylactic thrombolytic treatment received on same day as admission	2196	1532	(69.8)	67.8–71.7	33.2–84.6	A	RAND
Patient mobilized within 24 hours after surgery	1659	708	(42.7)	40.3–45.1	26.0–85.6	B	The Danish Clinical Registries
Patient with in-hospital surgical waiting time of <48 hours (or <2 days if time not provided)	2253	1248	(55.4)	53.3–57.5	35.2–84.4	C	OECD
**% of recommended care per case**	2288						
*No recommended care given*		150	(6.6)	5.5–7.6	1.2–23.7		
*25% of recommended care given*		640	(28.0)	26.1–29.8	10.4–44.0		
*50% of recommended care given*		796	(34.8)	32.8–36.7	23.7–44.7		
*75% of recommended care given*		568	(24.8)	23.1–26.6	14.4–44.2		
*100% of recommended care given*		134	(5.9)	4.9–6.8	0–18.1		
**Stroke (N = 2377)**							
Admitted directly to specialized stroke unit	2273	1013	(44.6)	42.5–46.6	5.1–81.4	A	The Danish Clinical Registries
Admitted to specialized stroke unit ≤24 hrs after hospital arrival	2261	1142	(50.5)	48.5–52.6	14.5–89.0	A	The Danish Clinical Registries
Treated with aspirin/antiplatelet ≤48 hrs after hospital arrival	2076	1948	(93.8)	92.8–94.9	88.0–96.8	A	The Danish Clinical Registries
CT OR MRI ≤24 hrs after hospital arrival	2249	2128	(94.6)	93.7–95.6	83.9–97.6	D	The Danish Clinical Registries
Patient mobilized within 48 hours or 2 days after admission	1617	1228	(75.9)	73.9–78.0	51.4–89.9	C/D	The Danish Clinical Registries
**Appropriate Stroke management (antiplatelet inhibitor within 48hours, CT or MRI performed within 24 hours and mobilized within 48 hours)**	1745	1012	(58.0)	55.7–60.3	35.8–82.7		

^1^Range of country-specific values for % Yes

(*) In bold the aggregate measures for each condition

At the hospital level, positive associations between quality management system and clinical indicators were found for only 4 of the 24 indicators analyzed: reperfusion therapy given in AMI (OR, 1.20; 95% CI 1.03 to 1.41), delivery of 75% of recommended care to hip fracture patients (OR, 1.17; 95% CI 1.03 to 1.33), admission to a specialized stroke unit (OR, 1.45; 95% CI 1.04 to 2.03) and administration of aspirin/antiplatelet drug within 48 hours of arrival at hospital (OR, 1.14; 95% CI 1.02 to 1.28). In this last case, a weak negative association was found with QMSI (OR, 0.94; 95% CI 0.88 to 1.00).

**Fig 2:**
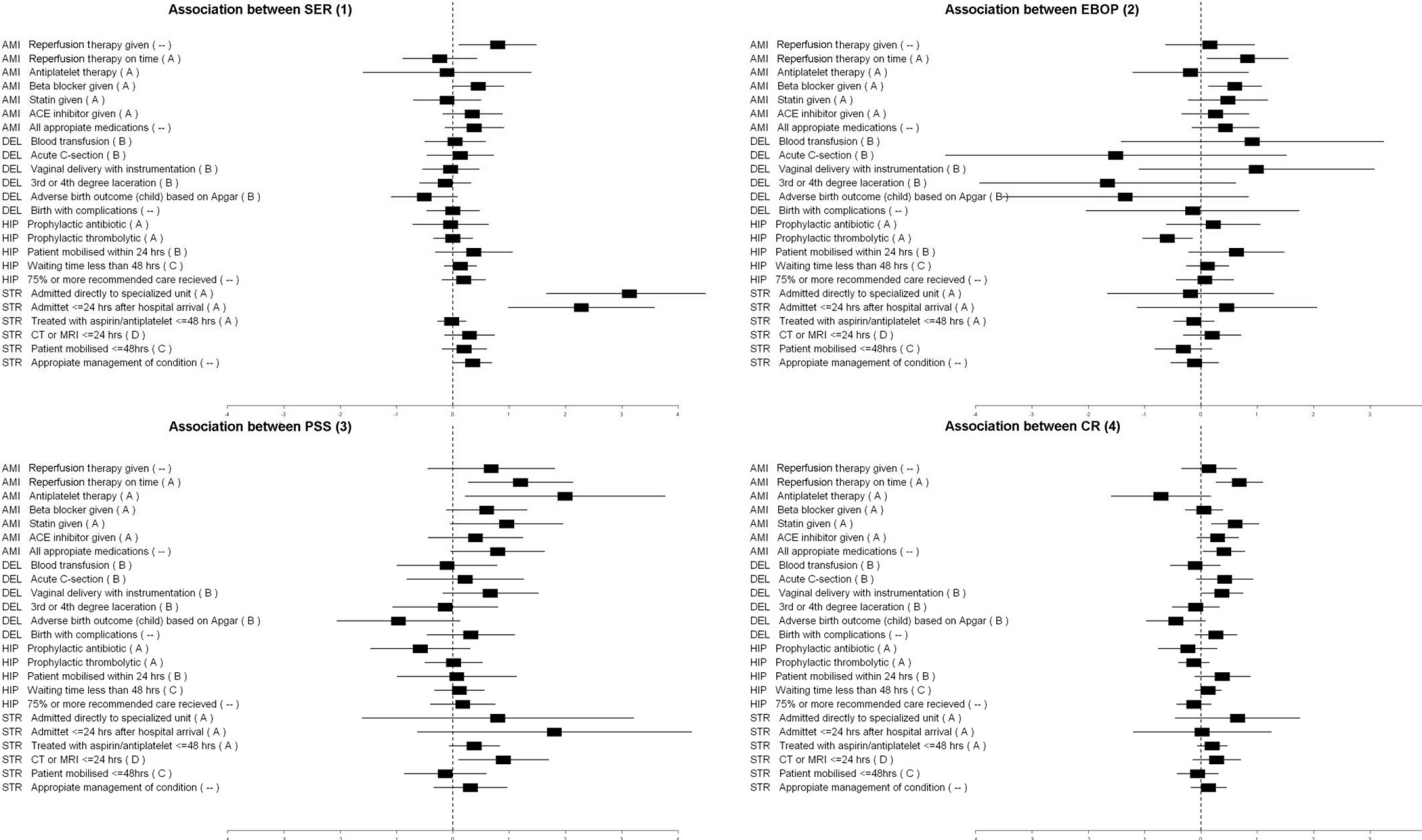
Clustered forest plot showing associations (OR and 95% CI) between department quality and clinical practice indicators, with level of evidence shown in brackets. Clustered forest plot showing associations (OR and 95% CI) between department quality and clinical practice indicators, with level of evidence shown in brackets. *(1) SER = Specialized expertise and responsibility; (2) EBOP = Evidence-based organization of pathways (EBOP); (3) = Patient Safety Strategies (PSS); (4) = Clinical Review*.

At the department level (Fig 2), we found substantially more significant associations between department-level quality activities and the clinical indicators analyzed, with a positive association observed for 50% of the indicators (12/24).

We also observed positive associations for a majority of the single indicators (5/6 indicators in AMI and 3/5 in stroke). Negative associations only were observed for one quality measure for 1 of 4 indicators for both deliveries and hip fracture. The percentage of positive associations between quality management systems and clinical indicators was substantially greater for indicators with level A evidence (7/10). Full table on the Association Between Hospital-Level Quality Measures and Clinical Practice Indicators is provided in Table A in S1 File; and full table on Association Between Department-Level Quality Measures (SER, PSS, and CR) and Clinical Practice Indicators in 4 Departments is provided in Table B in S2 File, both in the Supported Information files.

## Discussion

Our study is the largest of its kind in Europe to examine the impact of quality management on clinical practice. The results demonstrate wide variations in the implementation of hospital- and department-level quality management systems and in performance assessed by clinical practice indicators. Mean compliance with clinical practice indicators was 76.3%, demonstrating substantial room for improvement in EU hospitals. Previous studies have found similar variations in clinical practice based on the analysis of care received by both the general population [31–32] and hospitalized patients in different countries [33]. Our results for AMI were similar to average accomplishment rates reported by the GRACE study, a cohort study of outcomes for 9557 hospitalized patients with an acute coronary syndrome for the 2001–2007 period, but lower than the rates reported for 2007 [34]. This discrepancy could be linked to methodological differences, since the 2007 study was based on self-reporting by hospitals. Our study adds to the body of knowledge on variations in clinical practice by shedding light on associations between the implementation of quality management strategies and performance in clinical practice.

We found limited and weak associations between hospital-level quality management implementation and clinical indicators for all 4 conditions analyzed, but strong and clinically relevant associations for department-level quality management and clinical indicators for AMI and stroke. The observed effects remained strong and robust for both conditions after formal sensitivity analysis for uncontrolled confounding, selection bias, and measurement error using modern bias modeling methods [35–38] (results available from authors upon request). Reasons for the observed higher effectiveness of department quality activities could be linked to their proximity to the patient-provider interaction. Quality activities carried out close to the clinical decision level appear to have a greater impact on the implementation of evidence-based processes, leading to better clinical practice.

Our findings raise questions about the effectiveness of current quality efforts widely implemented in hospitals in high-income countries. Hospital programs should provide both methodological guidelines and support to departments to guide the design of quality programs, while continuing to focus on overall hospital responsibilities, such as policies, overall monitoring, and infection control.

Despite the recommendations of Shortell et al [39] in a study published over 15 years ago, hospital quality research is still largely focused on the impact of management at the hospital level. In a systematic review by Hearld et al in 2008 [40], over two-thirds of studies analyzed focused on hospital-level relationships, and in a recent literature review by our team [14], we found only 1 article that focused on quality management systems at the department level [41]. This predominant focus on hospital-level management systems is at odds with the increasing evidence that microsystems management has an important impact on clinical behavior and outcomes [42–44], with bottom-up organizational and clinical interventions applied in the front line appearing to lead to better results.

Why department-level quality measures seem to be more strongly associated with clinical indicators in the 2 medical pathways than in the surgical pathways could be linked to the type of care provided in these departments and/or to issues related to professional culture and practice, supporting the view that hospitals are loosely coupled systems whose components have limited or no knowledge of each other’s practices, resulting in little mutual influence and weak relationships [45–46]. This loosely coupled structure could be due to the tendency of hospitals to differentiate into subsystems with their own “laws” Top-down governance in hospitals seems to increasingly losing force with the enhancement of professional autonomy by the use of specific clinical practice guidelines that governs the organization of labor at the departmental level.

The above differences, however, could also be linked to the level of evidence of the indicators chosen and the adoption of evidence-based recommendations by departments [47–49]. In our study, positive associations were found between quality management activities at the department level for almost all indicators supported by level A evidence (7/10), which mostly applied to medical conditions. Furthermore, more and better-quality evidence is currently available for AMI and stroke than for vaginal deliveries and hip fracture surgery.

The findings of our study could also have an important impact on the organization of clinical departments. To evaluate the quality of activities at the department level, we choose 3 measures that were common to all departments (allocation of clinical responsibilities, implementation of patient safety strategies, and clinical review activities) and 1 specific measure (evidence-based organization of pathway), which while distinct for each condition, had the same structure in terms of patient flow (admittance, acute care, rehabilitation [if appropriate], and discharge). These 4 department-level quality measures seem to be associated with clinical practice indicators in AMI and stroke. Further studies are needed to investigate whether implementation of the above quality activities could be used to develop best practice recommendations applicable across hospital departments. If this were possible, a quality model for clinical departments combining department responsibilities, evidence-based organization, patient safety strategies, and quality review (audit and feedback) could be drawn up to provide guidance for clinical leaders when designing the organization of their departments.

## Limitations of the Study

This study has a number of limitations that need to be highlighted. First, we cannot draw conclusions on causality due to the cross-sectional nature of the study. Second, the medical records analyzed may have been more complete and comprehensive in some countries than in others [18]. Third, differences could be found in countries regarding regulatory requirements, national standards for quality, public reporting policies or other characteristics of the national health systems. We addressed this possible shortcoming by using a DAG to control for confounding in the development of our statistical models, incorporating theory and knowledge derived from previous research. We were therefore able to adjust for different country and hospitals characteristics in ways that allowed us to address competing explanations and plausible (non)causal associations, while minimizing sources of bias. We also used random-intercept mixed modeling with fixed effects to account for contextual features shared by hospitals within countries and for effects due to between-country differences. Further research and detailed measurements will be needed to tease out which specific characteristics might explain and modify between country variations. Other contextual factors from external and internal environment will need further studies in the future”. Another limitation is related to the hospital sampling strategy employed. Although sampling was random, generalization to participating countries and hospitals was limited because of possible self-selection by the hospitals that participated in the project. This is reflected in the different acceptance rates seen across the 7 countries analyzed. The reasons given for not participating in the study by hospitals in the countries with the lowest participation rates were related mainly to research fatigue, burnout with regard to quality management issues, time constraints, and competing interests with regard to efficiency and productivity targets. Additional limitations include the inclusion of multiple hypothesis tests, which, due to insufficient sample sizes, were not corrected for in our statistical analyses. Thus, the statistically significant relationships identified should be interpreted with caution.

## Conclusions

This study demonstrates significant gaps between evidence and practice for 4 common clinical conditions in a large sample of European hospitals. Our findings suggest that the implementation of department-level quality strategies is significantly associated with good clinical practice. Further research should aim to develop clinically relevant quality standards for hospital departments, as these appear to be more effective than the current widespread investment in generic, hospital-wide quality management systems.

## Supporting Information

S1 File (Table A). (DOCX)

S2 File (Table B). (DOCX)
