# Supplementary material for: Implementation of Departmental Quality Strategies Is Positively Associated with Clinical Practice: Results of a Multicenter Study in 73 Hospitals in 7 European Countries
Source: PLoS One. 2015 Nov 20;10(11):e0141157. doi: 10.1371/journal.pone.0141157 (PMC4654525; doi:10.1371/journal.pone.0141157)
Supplement: S1 File — (DOCX) [file pone.0141157.s001.docx]

**SUPPORTING INFORMATION FILE S1**

**Table A. Association Between Hospital-Level Quality Measures and Clinical Practice Indicators. Complete table.^6^**

| **Clinical Practice Indicators (Outcome variable)** | **HOSPITAL-LEVEL QUALITY MEASURES (Exposure variable)** | | |  |
| --- | --- | --- | --- | --- |
|  | **QMSI^1^**  ***Quality Management***  ***Systems Index*** | **QMSCI^1,2^**  ***Quality Management Compliance Index*** | **CQI^1,2,3^**  ***Clinical Quality***  ***Implementation Index*** | **Level of**  **Evidence** |
|  | **OR (LCL, UCL)** | **OR (LCL, UCL)** | **OR (LCL, UCL)** |  |
| **AMI** |  |  |  |  |
| Reperfusion therapy given | **1.20 (1.03, 1.41)** | 1.01 (0.81, 1.24) | 0.94 (0.73, 1.21) | -- |
| **Reperfusion therapy given on time** (fibrinolytic agent administered within 75 minutes of hospital arrival or primary percutaneous coronary intervention within 90 minutes) | 1.02 (0.88, 1.17) | 0.94 (0.76, 1.16) | 1.14 (0.92, 1.42) | A and A/B |
| Anti-platelet prescribed (or contraindicated) at discharge | 1.14 (0.93, 1.41) | 1.01 (0.71, 1.43) | 1.02 (0.70, 1.47) | A |
| Beta-blocker prescribed (or contraindicated) at discharge | 1.05 (0.95, 1.15) | 1.09 (0.94, 1.27) | 1.03 (0.88, 1.21) | A |
| Statin prescribed(or contraindicated) at discharge | 1.09 (0.95, 1.25) | 1.08 (0.88, 1.34) | 0.91 (0.73, 1.13) | A |
| ACE inhibitor prescribed (or contraindicated) at discharge | 1.02 (0.90, 1.15) | 1.12 (0.94, 1.33) | 0.94 (0.78, 1.13) | A |
| **All appropriate medications** (anti-platelet, beta-blocker, statin and ACE inhibitor) prescribed (or contraindicated) at discharge | 1.10 (0.98, 1.24) | 1.13 (0.95, 1.34) | 0.92 (0.76, 1.10) | -- |
| **Deliveries^4^** |  |  |  |  |
| Blood transfusion during vaginal birth | 0.95 (0.84, 1.08) | 1.05 (0.87, 1.27) | 0.90 (0.71, 1.15) | -- |
| Acute c-section | 0.96 (0.83, 1.11) | 0.98 (0.79, 1.23) | 0.91 (0.71, 1.17) | -- |
| Vaginal delivery with instrumentation | 0.92 (0.81, 1.03) | 0.91 (0.77, 1.08) | 0.93 (0.77, 1.12) | -- |
| Cases with third- or fourth-degree laceration | 1.06 (0.93, 1.22) | 1.09 (0.90, 1.32) | 1.03 (0.84, 1.26) | B |
| Adverse birth outcome (child): Apgar score <7 at 5 minutes | 1.09 (0.96, 1.25) | 0.91 (0.73, 1.12) | 1.03 (0.79, 1.33) | B |
| **Birth with complications (at least 1 positive indicator from above)** | 1.03 (0.92, 1.16) | 1.05 (0.88, 1.24) | 0.95 (0.78, 1.14) | -- |
| **Hip Fracture** |  |  |  |  |
| Prophylactic antibiotic within 60 minutes prior to surgical incision | 1.05 (0.93, 1.19) | 0.95 (0.78, 1.15) | 1.00 (0.80, 1.24) | A |
| Prophylactic thrombolytic treatment received on same day as admission | 0.99 (0.92, 1.06) | 0.93 (0.83, 1.04) | 1.02 (0.90, 1.16) | A |
| Patient mobilized within 24 hours | 0.96 (0.84, 1.11) | 1.04 (0.82, 1.31) | 1.20 (0.95, 1.51) | B |
| Patients with in-hospital surgical waiting time of < 48 hours (or <2 days if time not provided) | 1.04 (0.98, 1.11) | 1.08 (0.99, 1.19) | 1.06 (0.95, 1.17) | C |
| Delivery of <75% of recommended care | 0.96 (0.88, 1.05) | **1.17 (1.03, 1.33)** | 1.09 (0.95, 1.25) | -- |
| **Stroke** |  |  |  |  |
| Admitted directly to specialized stroke unit | **1.45 (1.04, 2.03)** | 0.87 (0.51, 1.48) | 1.06 (0.59, 1.90) | A |
| Admitted to specialized stroke unit ≤24 hours after hospital arrival | 1.35 (0.97, 1.88) | 1.16 (0.69, 1.96) | 1.33 (0.72, 2.47) | A |
| Treated with aspirin/antiplatelet ≤48 hours after hospital arrival | **0.94 (0.88, 1.00)** | 1.01 (0.91, 1.13) | **1.14 (1.02, 1.28)** | **A** |
| CT or MRI ≤24 hours after hospital arrival | 0.99 (0.89, 1.11) | 0.93 (0.78, 1.10) | 1.04 (0.84, 1.28) | D |
| Patient mobilized within 48 hours or 2 days | 0.95 (0.87, 1.05) | 0.89 (0.76, 1.03) | 1.03 (0.88, 1.22) | C/D |
| **Appropriate stroke management (antiplatelet inhibitor within 48 hours, CT or MRI within 24 hours, and mobilization within 48 hours)** | 0.94 (0.86, 1.02) | 0.95 (0.83, 1.09) | 1.06 (0.91, 1.23) | -- |

^1^Multivariate mixed logistic regression model with random intercept by hospital, and adjusted for fixed effects at the country level (country), hospital level (number of beds, teaching status, public vs. private), and patient level (age, gender).

^2^Further adjusted for hospital-level fixed effect of QMSI.

^3^Further adjusted for hospital-level fixed effect of QMSCI.

^4^Deliveries models do not include patient age as a covariate due to excessive missing data.

^5^Cumulative multinomial logistic model (OR per unit increase in % recommended care)

^6^ Significant results are shown in bold.
