# Supplementary material for: Implementation of Departmental Quality Strategies Is Positively Associated with Clinical Practice: Results of a Multicenter Study in 73 Hospitals in 7 European Countries
Source: PLoS One. 2015 Nov 20;10(11):e0141157. doi: 10.1371/journal.pone.0141157 (PMC4654525; doi:10.1371/journal.pone.0141157)
Supplement: S2 File — (DOCX) [file pone.0141157.s002.docx]

**SUPPORTING INFORMATION FILE S2**

**Table B. Association Between Department-Level Quality Measures (SER, PSS, and CR) and Clinical Practice Indicators in 4 Departments**

| **CLINICAL PRACTICE INDICATORS (Outcome variable)** | **DEPARTMENT-LEVEL QUALITY MEASURES (Exposure variable)** | | | | |
| --- | --- | --- | --- | --- | --- |
|  | **SER^1^**  ***Specialized expertise and responsibility*** | **EBOP^1,2^**  ***Evidence-based organization of pathways*** | **PSS^1^**  ***Patient Safety Strategies*** | **CR^1,2^**  ***Clinical Review*** | **Level of**  **Evidence** |
|  | **OR (LCL, UCL)** | **OR (LCL, UCL)** | **OR (LCL, UCL)** | **OR (LCL, UCL)** |  |
| **AMI** |  |  |  |  |  |
| Reperfusion therapy given | **2.21 (1.11, 4.41)** | 1.17 (0.53, 2.59) | 1.97 (0.64, 6.09) | 1.15 (0.70, 1.87) | -- |
| **Reperfusion therapy given on time** (fibrinolytic agent administered within 75 minutes of hospital arrival or primary percutaneous coronary intervention within 90 minutes) | 0.79 (0.41, 1.54) | **2.28 (1.11, 4.69)** | **3.32 (1.31, 8.43)** | **1.97 (1.30, 3.00)** | A and A/B |
| Anti-platelet prescribed (or contraindicated) at discharge | 0.90 (0.20, 3.98) | 0.83 (0.30, 2.35) | **7.31 (1.24, 43.08)** | 0.49 (0.20, 1.18) | A |
| Beta-blocker prescribed (or contraindicated) at discharge | **1.57 (1.00, 2.47)** | **1.82 (1.13, 2.92)** | 1.82 (0.89, 3.72) | 1.05 (0.75, 1.47) | A |
| Statin prescribed (or contraindicated) at discharge | 0.90 (0.49, 1.65) | 1.61 (0.79, 3.24) | 2.59 (0.95, 7.07) | **1.83 (1.20, 2.80)** | A |
| ACE inhibitor prescribed (or contraindicated) at discharge | 1.41 (0.83, 2.40) | 1.29 (0.71, 2.34) | 1.49 (0.64, 3.46) | 1.34 (0.92, 1.94) | A |
| **Appropriate medications** (anti-platelet, beta-blocker, statin and ACE inhibitor) prescribed (or contraindicated) at discharge | 1.46 (0.86, 2.46) | 1.54 (0.85, 2.80) | 2.21 (0.96, 5.12) | **1.50 (1.03, 2.18**) | -- |
| **Deliveries^3^** |  |  |  |  |  |
| Blood transfusion during vaginal birth | 1.04 (0.60, 1.77) | 2.48 (0.24, 25.4) | 0.90 (0.37, 2.19) | 0.90 (0.58, 1.42) | -- |
| Acute C-section | 1.14 (0.63, 2.07) | 0.22 (0.01, 4.26) | 1.24 (0.44, 3.52) | 1.52 (0.92, 2.52) | -- |
| Vaginal delivery with instrumentation | 0.96 (0.58, 1.59) | 2.67 (0.33, 21.6) | 1.94 (0.83, 4.55) | **1.45 (1.00, 2.10)** | -- |
| Cases with third- or fourth-degree laceration | 0.87 (0.55, 1.38) | 0.19 (0.02, 1.90) | 0.87 (0.34, 2.22) | 0.91 (0.60, 1.39) | B |
| Adverse birth outcome (child): Apgar score <7 at 5 minutes | 0.60 (0.33, 1.08) | 0.26 (0.03, 2.39) | 0.38 (0.13, 1.16) | 0.64 (0.38, 1.09) | B |
| **Birth with complications** (**at least 1 positive indicator from above**) | 1.00 (0.62, 1.59) | 0.86 (0.13, 5.73) | 1.37 (0.63, 2.99) | 1.30 (0.89, 1.89) | -- |
| **Hip fracture** |  |  |  |  |  |
| Prophylactic antibiotic within 60 minutes prior to surgical incision | 0.96 (0.49, 1.89) | 1.24 (0.54, 2.86) | 0.56 (0.23, 1.35) | 0.79 (0.47, 1.34) | A |
| Prophylactic thrombolytic received on same day as admission | 1.00 (0.70, 1.42) | 0.55 (0.35, 0.85) | 1.01 (0.60, 1.68) | 0.88 (0.67, 1.16) | A |
| Patient mobilized within 24 hours (*n*= 1572) | 1.45 (0.73, 2.89) | 1.87 (0.80, 4.37) | 1.07 (0.37, 3.10) | 1.46 (0.89, 2.41) | B |
| Patients with in-hospital surgical waiting time <48 hours (or <2 days if time not provided ) | 1.14 (0.86, 1.53) | 1.12 (0.77, 1.64) | 1.12 (0.72, 1.76) | 1.13 (0.89, 1.43) | C |
| Delivery of <75% of recommended care | 1.21 (0.82, 1.78) | 1.07 (0.64, 1.77) | 1.19 (0.67, 2.13) | 0.88 (0.65, 1.20) | --- |
| **Stroke** |  |  |  |  |  |
| Admitted directly to specialized stroke unit | **22.85 (5.23, 99.92)** | 0.83 (0.19, 3.64) | 2.21 (0.20, 25.01) | 1.91 (0.63, 5.78) | A |
| Admitted to specialized stroke unit ≤24 hours after hospital arrival | **9.75 (2.65, 35.85)** | 1.58 (0.32, 7.83) | 6.05 (0.53, 68.83) | 1.02 (0.30, 3.49) | A |
| Treated with aspirin/antiplatelet ≤48 hours after hospital arrival | 0.98 (0.76, 1.27) | 0.88 (0.61, 1.26) | 1.46 (0.93, 2.29) | 1.22 (0.93, 1.59) | A |
| CT OR MRI <24 hours after hospital arrival | 1.34 (0.86, 2.10) | 1.22 (0.73, 2.04) | **2.45 (1.10, 5.49)** | 1.32 (0.86, 2.03) | D |
| Patient mobilized within 48 hours or 2 days | 1.22 (0.82, 1.82) | 0.73 (0.44, 1.21) | 0.87 (0.42, 1.81) | 0.94 (0.65, 1.36) | C |
| **Appropriate stroke management** | 1.42 (1.01, 2.00) | 0.89 (0.58, 1.37) | 1.36 (0.71, 2.63) | 1.14 (0.83, 1.57) | -- |

^1^Multivariate mixed logistic regression model with random intercept by hospital, adjusted for fixed effects at the country level (country), hospital level (number of beds, teaching status, public vs. private, QMSI, CQI, QMSCI), and patient level (age, gender).

^2^Additionally adjusted for department level fixed effect: quality measure SER.

^3^Deliveries models do not include patient age as a covariate due to excessive missing data.

^5^Cumulative multinomial logistic model (OR per unit increase in % recommended care)

^6^ Significant results are shown in bold.
